# Egg yolk-derived phosvitin as a natural chelator: *In vivo* mitigation of mercury toxicity in rats

**DOI:** 10.14202/vetworld.2025.2900-2917

**Published:** 2025-09-30

**Authors:** Made Sriasih, Anwar Rosyidi, Rukmanggana Satya Pratiwi, I Gusti Ayu Sri Andayani, Citranggana Prajnya Dewi, Ardiana Ekawanti, Ryan Aryadin Putra, Sulaiman Ngongu Depamede

**Affiliations:** 1 Laboratory of Biotechnology and Animal Products, Faculty of Animal Science, University of Mataram, West Nusa Tenggara, Indonesia; 2 Medical Study Program, Faculty of Medicine and Health Sciences, University of Mataram, West Nusa Tenggara, Indonesia; 3 Laboratory of Animal Nutrition and Feed Science, Faculty of Animal Science, University of Mataram, West Nusa Tenggara, Indonesia

**Keywords:** egg yolk, mercury toxicity, natural chelator, oxidative stress, phosvitin, rat

## Abstract

**Background and Aim:**

Mercury (hydrargyrum, Hg) exposure poses significant risks to human, animal, and environmental health due to its persistence and toxicity. Conventional chelating agents, though effective, are limited by adverse side effects and reduced efficacy in chronic exposure. Phosvitin (PSV), a highly phosphorylated protein from egg yolk with strong metal-binding capacity, offers potential as a natural detoxifying agent. This study aimed to evaluate, for the first time *in vivo*, the chelating efficacy of egg yolk-derived PSV against Hg toxicity in rats.

**Materials and Methods:**

PSV was isolated from Isa Brown hen egg yolks through ethanol precipitation and characterized for purity and antioxidant activity using Fourier Transform Infrared Spectroscopy, Sodium Dodecyl Sulfate–Polyacrylamide Gel Electrophoresis, Kjeldahl analysis, and the 2,2-diphenyl-1-picrylhydrazyl assay. Twenty-five male rats were exposed to Hg-contaminated fish feed (0.17 ppm for 14 days) and subsequently treated with commercial or isolated PSV at 10, 20, or 30 ppm for 4 weeks. Hematological indices, liver and kidney histopathology, and Hg accumulation in brain tissue were assessed. Data were analyzed using R software (v4.4.0), with p < 0.05 considered statistically significant.

**Results:**

The isolated PSV exhibited moderate antioxidant activity, comparable to that of commercial standards, with a purity of 51.68%. Hg exposure significantly increased white blood cell (WBC) and basophil counts, indicating immune activation. PSV administration, particularly at 20 ppm, markedly reduced WBC and basophil levels, reflecting immunomodulatory activity. Histopathological analysis revealed reduced hepatic necrosis and milder liver damage in treated groups, with modest improvement in renal structure, though not statistically significant. Brain analysis showed decreased Hg accumulation in the 10 and 20 ppm PSV groups, while the 30 ppm group exhibited inconsistent effects.

**Conclusion:**

Egg yolk-derived PSV, especially at 20 ppm, effectively mitigated Hg-induced hematological, hepatic, and neural toxicity, supporting its role as a safe natural chelator. These findings highlight its potential as a dietary intervention in Hg-exposed populations. Optimization of extraction methods, larger-scale studies, and long-term safety assessments are needed to advance its translational application in toxicology and public health.

## INTRODUCTION

Mercury (hydrargyrum, Hg) is a highly toxic heavy metal [1] that occurs in several forms, including elemental mercury (Hg^°^), methylmercury (MeHg), inorganic mercury (Hg^2+^), and various organic compounds [2]. Under normal conditions, it is volatile and can be released into the atmosphere through both natural processes and anthropogenic activities, with human sources contributing approximately 2,000–2,500 tons annually [1–3]. Among these, artisanal and small-scale gold mining (ASGM) is a dominant contributor, accounting for nearly 37.5% of global Hg emissions and releasing up to 1,000 tons each year [1, 4, 5]. During ASGM, liquid Hg^°^ is oxidized to Hg(II), which subsequently undergoes methylation to form the highly toxic MeHg [2, 6]. In addition, inhalation risk is elevated through Hg vapor emitted during amalgam burning. Even at low concentrations, exposure through contaminated food, water, or air poses serious health risks across species.

In humans, acute high-dose exposure typically results in gastrointestinal disturbances, whereas chronic exposure leads to gingivitis, tremors, and erethism [6]. Severe poisoning may cause cognitive impairment, motor dysfunction, and, in extreme cases, death [2]. Vulnerable groups include pregnant women, fetuses, and immunocompromised individuals, who face elevated risks of cancer and developmental toxicity. Maternal exposure can cause congenital malformations, miscarriage, low birth weight, and stillbirth [7]. Long-term Hg exposure has also been associated with cardiotoxicity, reproductive impairments, renal dysfunction, and cancer development [2]. In livestock, poisoning manifests as neurological and gastrointestinal disturbances, while chronic exposure compromises immunity, organ integrity, and productivity [8].

Clinical management of Hg intoxication primarily relies on chelation therapy [2, 9] using synthetic agents such as penicillamine, dimercaprol (British anti-Lewisite, BAL), meso-2,3-dimercaptosuccinic acid (succimer, DMSA), and 2,3-dimercapto-1-propanesulfonic acid (unithiol, DMPS). These compounds facilitate Hg excretion by forming stable complexes [2]. While effective in acute poisoning, their efficacy in chronic exposure is limited, largely due to poor mobilization of intracellular Hg [9, 10]. Moreover, these chelators are associated with adverse effects, including hepatotoxicity, nephrotoxicity, and cardiovascular complications [11]. These shortcomings highlight the urgent need for safer, sustainable alternatives.

Natural biomolecules with strong and versatile metal-binding properties offer promising solutions [12]. Phosvitin (PSV), a highly phosphorylated egg yolk protein, possesses abundant phosphoserine residues that coordinate with divalent metal ions through electron donation, thereby forming soluble complexes and mitigating metal-induced oxidative stress [13–15]. Compared with other natural chelators, such as casein and ferritin, PSV exhibits superior metal-binding efficiency due to its dense phosphorylation, digestive stability, and enhanced antioxidant activity under physiological conditions [16–18]. Its wide availability, low cost, and nutritional origin further support its potential for therapeutic and environmental applications.

PSV’s metal-binding activity is particularly well established for iron, sequestering nearly 95% of yolk iron [19] with a strong ferric ion binding constant (10^7^–10^18^) [20]. It remains stable under high temperature and pressure [14], and its capacity to inhibit hydroxyl radical formation through iron chelation underscores its potent antioxidant function [21, 22].

Despite extensive studies on synthetic chelating agents such as DMSA, DMPS, and BAL, their clinical use is restricted by toxicity, poor mobilization of intracellular Hg, and limited long-term efficacy. Natural biomolecules have been explored as alternatives, but most prior studies have been confined to *in vitro* or biochemical assays, with scarce evidence from controlled *in vivo* systems. PSV, one of the most phosphorylated proteins in nature, is well recognized for its strong affinity toward divalent and trivalent metals, including iron and calcium. While its iron-chelating, antioxidant, and nutraceutical properties are established, little is known about its ability to mitigate heavy metal toxicity, particularly Hg, in living organisms. No comprehensive animal model studies have directly evaluated whether egg yolk–derived PSV can reduce Hg accumulation in tissues, restore hematological balance, or alleviate histopathological damage. This lack of experimental validation represents a major gap in translating PSV from a food-derived protein into a potential therapeutic candidate for heavy metal detoxification.

This study was designed to address these knowledge gaps by evaluating, for the first time *in vivo*, the chelating efficacy of PSV against Hg-induced toxicity. Specifically, the research aimed to: (i) isolate and characterize PSV from egg yolk and determine its antioxidant potential; (ii) assess the effects of PSV supplementation on hematological parameters in rats exposed to Hg; (iii) examine liver and kidney histopathology following treatment; and (iv) quantify Hg accumulation in brain tissue to elucidate its detoxifying potential. Through this integrated approach, the study aims to determine whether egg yolk–derived PSV can serve as a safe, natural alternative to synthetic chelators, thereby contributing to the development of nutritionally based interventions for managing Hg exposure.

## MATERIALS AND METHODS

### Ethical approval

The ethical approval was granted by The Health Research Ethics Committee, Faculty of Medicine and Health Sciences, University of Mataram (Approval No. 064/UN18.F8/ETIK/2024). In accordance with the Animal Research: Reporting of *In Viv*o Experiments (ARRIVE 2.0) guidelines, ethical approval for this *in vivo* study was granted by the institutional ethics committee following a thorough review of our research proposal. This review ensured that the study met established standards for transparency, reproducibility, and ethical conduct in the use of animals for scientific research. All procedures were designed to uphold animal welfare and scientific integrity throughout the experimental process.

### Study period and location

This study was conducted from February to November 2024. Isolation and characterization were carried out at the Microbiology and Biotechnology Laboratory, Faculty of Animal Science, University of Mataram. *In vivo* study was performed at the Drug Testing Laboratory, Pharmacy Study Program, Faculty of Medicine and Health Sciences, University of Mataram.

### Isolation of PSV from layer egg yolk

PSV was isolated using a modified method described by Ko *et al*. [23]. Fresh eggs from Isa Brown hens were sourced locally from a semi-intensive farm in Lombok, West Nusa Tenggara, Indonesia. Hens were maintained on a semi-intensive system and fed a standard diet comprising maize, concentrate, bran, and mineral supplements. Egg yolks were manually separated, and 150 g were homogenized with two volumes of distilled water until uniform. The pH of the homogenate was adjusted to 7.0 using 6 N hydrogen chloride (HCl) (Merck, Germany). The mixture was centrifuged at 3,220 × *g* for 30 min at 4°C. The supernatant was discarded, and the precipitate was retained for subsequent processing. The pellet was resuspended in four volumes of 85% ethanol (Merck) and homogenized for 2 min. The ethanol-treated sample was centrifuged at 3,220 × *g* for 10 min. The resulting sediment was homogenized and mixed with nine volumes of 10% sodium chloride (NaCl) (Merck). The pH of the mixture was then adjusted to 4.0 using 1 M HCl. A final centrifugation was performed at 3,220 × *g* for 30 min at 4°C. The supernatant was filtered through the Whatman No. 1 filter paper (Whatman, UK) to eliminate insoluble residues. The filtrate was dialyzed in 0.9% NaCl buffer using a 10 kDa MWCO cellulose membrane (Viskase Sales Corp., USA) for 24 h at 4°C, with two buffer replacements. The dialysate was concentrated at 4°C using ultrafiltration (GE Healthcare Bio-Sciences Corp., NJ, USA) and then lyophilized at −40°C under 220 mbar pressure. Lyophilized PSV was stored at −20°C until further analysis.

### PSV antioxidant activity assay

The antioxidant activity of isolated PSV was determined using the 2,2-diphenyl-1-picrylhydrazyl (DPPH) assay (Sigma-Aldrich, Germany) following the method described by Hussen and Endalew [24]. A 100 ppm DPPH stock solution was prepared by dissolving 5 mg of DPPH in 50 mL of methanol (Merck). The absorbance of the solution was measured spectrophotometrically to confirm stability. Serial dilutions of the DPPH solution yielded concentrations of 10, 20, 30, 40, and 50 ppm. To ensure homogeneity, each diluted solution was stored at room temperature for 24 h. Ascorbic acid (Sigma-Aldrich) was used as a positive control and was subjected to identical testing conditions. Ascorbic acid was chosen as a comparator based on its reputation as a compound with high antioxidant activity.

Both control and test sample solutions were prepared separately for the assay. The control consisted of 2 mL of methanol and 1 mL of DPPH stock solution. Each test sample consisted of 2 mL of PSV solution at a given concentration mixed with 2 mL of DPPH solution. Samples were incubated at 27°C for 30 min in the dark. This incubation allowed the DPPH radicals to react with the antioxidant compounds present in the sample. A visible color change indicates successful radical scavenging activity. After incubation, 300 μL of each sample was transferred to a microplate, and the absorbance was measured at 517 nm using a Multiscan Sky spectrophotometer (Thermo Fisher Scientific, USA). To ensure reproducibility, each test was performed in triplicate. The percentage inhibition values were plotted against the concentration to calculate the half maximal inhibitory concentration (IC_50_), defined as the concentration required to inhibit 50% of DPPH radicals.


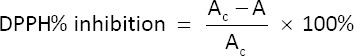


A_c_ = mean absorbance value of the control

A = mean absorbance value of the sample

### Classification of antioxidant strength

Compounds with an IC_50_ below 50 ppm are considered very strong antioxidants. An IC_50_ of 50–100 ppm is classified as strong, 101–250 ppm as moderate, and 250–500 ppm as weak [25].

### Fourier transform infrared spectroscopy (FTIR)

FTIR spectroscopy is a powerful analytical technique used to identify molecular structures. This technique was used to identify and confirm the groups of phosphorylated glycoproteins from egg yolk. One milligram of the isolated PSV was finely ground with potassium bromide (KBr) (Merck) to obtain a homogeneous mixture. The mixture was compressed into a pellet approximately 1 mm thick using a pellet press. The pellet was placed in the sample holder, and absorbance spectra were recorded from 400 cm^−1^ to 4000 cm^−1^ at a resolution of 4 cm^−1^ using a PerkinElmer FTIR spectrophotometer. The resulting spectrum displayed characteristic absorption peaks corresponding to functional groups, plotted as wavenumber versus percentage transmittance. The sample spectrum was compared with that of commercial PSV (Sigma-Aldrich) to confirm structural similarity.

### Sodium dodecyl sulfate–polyacrylamide gel electrophoresis (SDS-PAGE)

One-dimensional SDS-PAGE was used to assess the molecular weight and purity of PSV by separating proteins based on size under denaturing conditions. This method effectively confirms the presence of PSV, detects potential impurities or degradation products, and ensures consistent quality. Briefly, SDS-PAGE analysis was performed using a 10% FastCast Gel (Bio-Rad, USA). All electrophoresis reagents were obtained from Bio-Rad (USA). Gels were prepared using TGX Acrylamide Solutions following standard protocols. PSV samples were mixed with 2× Laemmli buffer and β-mercaptoethanol before denaturation. Samples were denatured by heating at 95°C for 5 min and then cooled on ice. Each sample (25 μL) was loaded into the gel wells. A Precision Plus Protein Stain-Free molecular weight marker was used as a reference. Electrophoresis was performed in a Mini-Protean Tetra Vertical Electrophoresis Cell at 150 V for 40 min. Tris-glycine-SDS buffer was used as the running buffer. Protein bands were visualized using the stain-free method and analyzed using the GelDoc Go Imaging System (Bio-Rad).

### Quantitative analysis of PSV purity (Kjeldahl method)

Exactly 0.2583 g of isolated PSV was weighed for nitrogen analysis. The nitrogen content was determined using the Kjeldahl method with a Behrotest analyzer (Merck). Titration was performed using 0.1 N sulfuric acid (H_2_SO_4_) (Merck) as the titrant. The sample titration volume and the corresponding blank were recorded to calculate the nitrogen concentration. The nitrogen content was calculated using the standard Kjeldahl conversion formula.


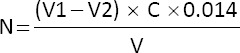


V = sample weight (0.2583 g)

V1 = sample titration volume

V2 = control volume

C = 0.1 N (H_2_SO_4_ concentration)

0.014 = Kjeldahl conversion factor

The protein content was estimated by multiplying the nitrogen percentage by a PSV-specific conversion factor of 7.69 [26].

### *In vivo* experimental animals and study design

Twenty-five adult male rats (*Rattus norvegicus*), each weighing approximately 200 g, were housed in a temperature-controlled room (21°C–25°C). The photoperiod was maintained under a 12-h light/12-h dark cycle. Rats were randomly assigned to five groups, with five animals per cage. All rats underwent a 1-week acclimatization period before treatment initiation. Animals were fed a commercial pelleted diet and had water available *ad libitum* throughout the study. Following acclimatization, all rats received 0.17 ppm Hg-contaminated fish feed through oral gavage daily for 14 days. The Hg levels in the fish feed were pre-confirmed by Cold Vapor Atomic Absorption Spectrophotometry (Mercury Instruments GmbH, Germany). After 14 days of Hg exposure, the rats received different PSV treatments as follows: PN (negative control, no PSV), PP (positive control, 4 ppm commercial PSV), PV10 (10 ppm isolated PSV), PV20 (20 ppm), and PV30 (30 ppm). PSV was administered by oral gavage once daily for 4 consecutive weeks. Feed and water were provided *ad libitum* during the treatment. A completely randomized design was used to evaluate the chelating effects of PSV on Hg-exposed rats.

### Sample collection and hematological examination

Blood samples were collected through tail vein puncture before and after exposure to Hg. At the endpoint of the study, the rats were weighed and euthanized with an intraperitoneal injection of ketamine (80 mg/kg body weight) (PT Bernofarm, Indonesia) and xylazine (10 mg/kg body weight) (Xyla Interchemie, The Netherlands). The loss of eyelid and pedal withdrawal reflexes was monitored to confirm adequate anesthesia. Cardiac puncture was performed to obtain whole blood samples during terminal anesthesia. Blood was transferred into ethylenediaminetetraacetic acid-coated tubes (PT Endo Indonesia, Indonesia) to prevent coagulation, and the tubes were gently inverted to ensure proper mixing of the anticoagulant. Complete blood counts were performed to measure white blood cells (WBCs), leukocyte subpopulations (neutrophils, eosinophils, basophils, monocytes, and lymphocytes), red blood cells (RBCs), hemoglobin (Hb), hematocrit (HCT), and platelet levels. Hematological analysis was performed using a Sysmex XP-300 automated analyzer (Sysmex Corporation, Japan).

### Histopathological evaluation of liver and kidney

Following blood collection, liver, kidney, and brain tissue were excised for further analysis. Liver and kidney tissue samples were fixed overnight in 10% neutral-buffered formalin (Merck). The fixed organs were stored in 70% ethanol and submitted for routine histological analysis to the Department of Anatomical Pathology, Faculty of Medicine, Airlangga University, East Java, Indonesia. To maintain objectivity, all samples were labeled with anonymized codes before submission to conceal the treatment group identities. The anatomical pathologist conducting the evaluation remained blinded to the experimental conditions throughout the study. Tissues were embedded in paraffin, sectioned at 3 μm–5 μm with a microtome, mounted on glass slides, and stained with Hematoxylin and Eosin (H&E). The stained slides were cleared in xylene and mounted with coverslips. Histological evaluation was performed using an Olympus CX41RF binocular microscope (Olympus, Japan). Five random fields per section were examined at 400× magnification to assess pathological changes. The renal pathology assessment included normal tubules, tubular necrosis, degeneration, and inflammatory cell infiltration. Hepatic tissue was evaluated for hepatocyte integrity, inflammation, necrosis, and hydropic degeneration. Tissue damage was graded on a 0–4 scale [27]: 0 = none, 1 = mild (focal), 2 = moderate (<25%), 3 = severe (25%–50%), and 4 = very severe (>50%).

### Brain tissue heavy metal analysis

Brain tissue was preserved in tubes containing phosphate-buffered saline (PBS) (pH 7.2) and frozen at −20°C for later analysis of heavy metal content. The frozen samples were thawed, and 10 mg of brain tissue was transferred to microcentrifuge tubes. The samples were homogenized in 100 μL of PBS using a handheld ultrasonic homogenizer for 2 min. Homogenates were freeze-dried at −40°C and 220 mbar for 24 h using a Christ Alpha 1-2 LDplus (Germany) freeze dryer. Lyophilized brain samples were analyzed using a scanning electron microscope equipped with energy-dispersive X-ray spectroscopy (JEOL JCM-7000, JEOL Ltd., Japan), with magnifications up to 100,000×.

### Statistical analysis

Statistical analysis was performed using the R software (v4.4.0) with the “agricolae” package [28]. A p < 0.05 was considered statistically significant. The applied model was:

y_ij_ = μ + τ_i_ + ɛ_ij_,

where y_ij_ = the observed value of each individual, μ = the overall mean, τ_i_ = the treatment effect, and ɛ_ij_ = random error.

## RESULTS

### Isolation and characterization of PSV

#### Physical properties and antioxidant activity

In this study, PSV isolated from the egg yolk layer, following the method described by Ko *et al*. [23], exhibited physical characteristics as a fine yellowish powder. Its antioxidant activity, as depicted in Table 1, demonstrated a moderate antioxidant capacity with a value of 124.85 ppm. Similarly, commercial PSV exhibited a comparable level of antioxidant activity (125.27 ppm). In contrast, ascorbic acid, used as a reference control, showed a significantly stronger antioxidant effect, indicating its stronger radical scavenging potential compared with both PSV samples.

**Table 1:** Antioxidant levels of phosvitin isolated from layer egg yolk.

Sample	IC_50_ (ppm)	Antioxidant activity
Isolated phosvitin	124.85	Medium
Commercial phosvitin	125.27	Medium
Ascorbic acid	7.06	Very strong

IC_50_ = Half maximal inhibitory concentration

#### FTIR spectral analysis

**Figure 1:**
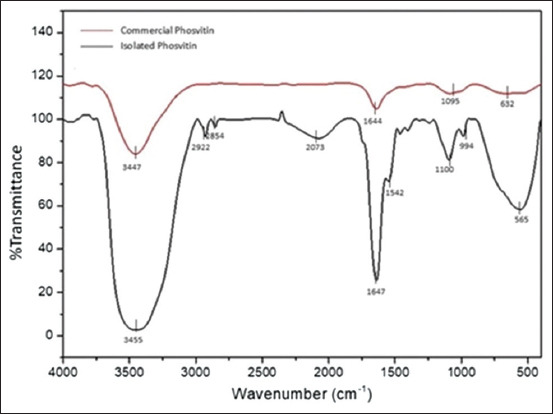
Fourier transform infrared spectroscopy spectra of isolated and commercial phosvitin.

The qualitative purity analysis of the isolated PSV sample and the standard commercial PSV, conducted using the KBr disc FTIR method, revealed similar spectral patterns. Figure 1 shows the recorded spectra for both the sample and the standard obtained through FTIR analysis.

Figure 1 shows the percentage transmittance (%Transmittance) at various wavenumbers (cm^−1^), illustrating the presence and interaction of the functional groups. The spectral peak observed at 3500 cm^−1^–3000 cm^−1^ indicates the presence of hydroxyl (–OH) groups associated with hydrogen bonding. In addition, the peak around 1650 cm^−1^ corresponds to the C=O bond, which is a characteristic of Amide I, which is commonly found in protein structures. Another peak at approximately 1550 cm^−1^ is linked to Amide II vibrations, confirming the presence of amide groups involving N–H bending and C–N stretching within the PSV peptide sequence. The 1650 cm^−1^– 1550 cm^−1^ range reflects protein conformation and bonding within secondary and tertiary structures.

Minor shifts in peak positions during the isolation process may indicate variations in protein interactions or denaturation. At approximately 1100 cm^−1^–1000 cm^−1^, the spectrum exhibits a peak corresponding to phosphate groups (PO_4_^3−^), a key feature of PSV that plays a crucial role in mineral binding, particularly with metal ions such as calcium, iron, and phosphorus. The range of 600 cm^−1^–500 cm^−1^ is associated with P–O vibrations, representing interactions between phosphate groups and metal ions, including ferric ion (Fe^3+^) and calcium ion (Ca^2+^). The obtained FTIR spectrum supports the presence of PSV in the isolated sample, as evidenced by the distinctive amide and phosphate peaks. However, additional analyses, such as SDS-PAGE or other protein assays, are required to validate the FTIR results.

#### SDS-PAGE purity analysis

**Figure 2:**
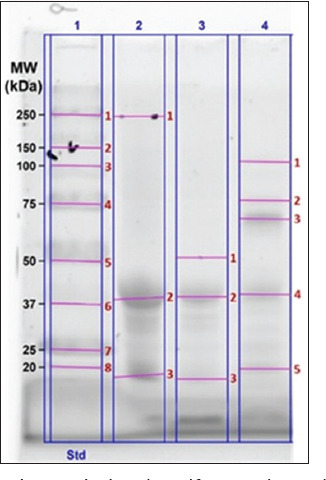
Sodium dodecyl sulfate–polyacrylamide gel electrophoresis profile of phosvitin isolated from the egg yolk layer. Lane 1: Precision Plus protein molecular weight marker standard. Lane 2: Commercial phosvitin (8× dilution). Lane 3: Commercial phosvitin (16× dilution). Lane 4: isolated phosvitin. The numbers in red indicate the protein bands with specific molecular weights.

The qualitative purity analysis of isolated PSV, performed using SDS-PAGE and presented in Figure 2, indicates that commercial PSV exhibits higher purity compared to isolated PSV, as assessed by the number of protein bands observed. The commercial PSV sample displayed three protein bands, whereas the isolated PSV sample exhibited five protein bands, suggesting the presence of additional protein components in the isolated sample.

The molecular weight of the protein bands on the gel was automatically detected and measured with high sensitivity using the GelDoc Go Imaging System’s automated molecular weight analysis software. Only protein bands within the molecular weight range of 250 kDa–20 kDa, consistent with the molecular weight standard, were analyzed. Figure 2 and Table 2 show that a dominant (thick) protein band was observed in all commercial and isolated PSV samples within the 39.5 kDa–38.5 kDa range.

**Table 2:** Molecular weight of protein bands in isolated and commercial phosvitin samples.

Band number	Molecular weight (kDa)			

	Lane 1	Lane 2	Lane 3	Lane 4
1	250.0	243.6	51.2	110.3
2	150.0	38.5	38.9	76.9
3	100.0	20.0	20.0	67.4
4	75.0			39.5
5	50.0			20.0
6	37.0			
7	25.0			
8	20.0			

#### Quantitative protein purity (Kjeldahl method)

The quantitative analysis of PSV purity using the Kjeldahl method revealed that the nitrogen content in the analyzed sample was 0.0672 g, with a blank volume of 0 mL, assuming that the volume of solution required for sample titration was 12.4 mL. Based on the specific protein conversion factor for PSV (7.69), the purity of PSV in the sample was determined to be 51.68%. This result indicates that more than half of the sample’s composition consists of pure PSV, while the remaining portion likely includes non-protein compounds such as lipids, carbohydrates, or other residual substances from the extraction process.

### *In vivo* study

#### Hematological profiles

Table 3 shows the impact of Hg exposure on the hematological profiles of the test rats. After 2 weeks of Hg-contaminated fish consumption, the WBC count, a primary indicator of immune response, increased by 41.03%, rising from 10.26 ± 3.27 × 10^9^/L to 14.47 ± 5.96 × 10^9^/L. In addition, changes were observed in the distribution of WBC subpopulations, with neutrophils decreasing by 50.50% and lymphocytes increasing by 5.21%, from 66.47 ± 7.28 to 69.93 ± 10.98. Notable increases were also detected in monocytes (464.39%) and eosinophils (172.41%). Moreover, basophils exhibited a striking increase of 3,672.73%, rising from 0.11 ± 0.32% to 4.15 ± 2.75%.

**Table 3:** Pre- and post-mercury hematological values (mean ± standard deviation) 2 weeks after mercury-contaminated fish administration in test rats.

Variable	Pre-mercury	Post-mercury
WBCs (×10^9^/L)	10.26 ± 3.27	14.47 ± 5.96
Neutrophil (%)	30.95 ± 7.23	15.32 ± 7.8
Lymphocyte (%)	66.47 ± 7.28	69.93 ± 10.98
Monocyte (%)	1.32 ± 0.82	7.45 ± 12.83
Eosinophil (%)	1.16 ± 1.21	3.16 ± 2.67
Basophil (%)	0.11 ± 0.32	4.15 ± 2.75
Hb (g/dL)	16.11 ± 0.96	15.98 ± 1.66
RBCs (×10^6^/µL)	8.18 ± 0.52	9.31 ± 0.94
HCT (%)	41.05 ± 3.99	46.78 ± 4.87
Platelet (×10^3^/µL)	609.42 ± 71.31	539.32 ± 326.90

WBCs = White Blood Cells, Hb = Hemoglobin, RBCs = Red blood cells, HCT = Hematocrit

Despite the marked changes in WBC components, the influence of Hg exposure on Hb levels appeared to be relatively minor, with only a 0.81% reduction, from 16.11 ± 0.96 g/dL to 15.98 ± 1.66 g/dL. Conversely, RBC count and HCT levels showed increases of 13.81% and 13.96%, respectively, while platelet count decreased by 11.50%.

The hematology profile indicates that PSV treatment has a significant effect on WBC and basophil concentrations in rat models exposed to Hg (Table 4). PSV treatments resulted in a notable reduction in WBC counts compared with PN. The PN exhibited the highest WBC value of 12.84 × 10^9^/L, which was significantly different from the PP of 8.48 × 10^9^/L, as well as from all PSV treatments (PV10, PV20, and PV30) (p = 0.001). The lowest WBC concentration was observed in PV30, with a value of 5.82 × 10^9^/L. This decline in the number of WBCs suggests that PSV may modulate the immune system in rats by reducing inflammatory responses or immunological stress.

**Table 4:** Effects of 4-week phosvitin administration on the hematological values of rats fed with mercury-contaminated fish.

Variable	Treatments					SEM	p-value

	PN	PP	PV10	PV20	PV30		
WBCs (×10^9^/L)	12.84^a^	8.48^b^	6.68^b^	8.40^b^	5.82^b^	0.361	0.001
Neutrophil (%)	32.15	34.50	38.50	21.88	25.04	2.352	0.164
Lymphocyte (%)	56.25	56.30	50.20	64.88	55.86	2.251	0.314
Monocyte (%)	3.60	5.47	6.64	11.52	7.83	1.198	0.372
Eosinophil (%)	0.70	1.10	0.80	0.70	0.70	0.087	0.479
Basophil (%)	4.80^a^	7.30^ab^	3.53^bc^	0.62^c^	1.78^bc^	0.048	0.014
Hb (g/dL)	14.85	14.30	12.85	13.28	12.90	0.264	0.223
RBCs (×10^6^/µL)	8.75	8.30	7.69	7.97	7.49	0.159	0.244
HCT (%)	46.90	44.23	41.57	43.90	40.80	0.883	0.353
Platelet (×10^3^/µL)	844.50	776.00	903.67	694.00	660.20	49.904	0.493

Different letters (a–c) within rows indicate significant differences between treatments (p < 0.05). PN = Negative control, PP = Positive control, PV10 = Phosvitin at 10 ppm, PV20 = Phosvitin at 20 ppm, PV30 = Phosvitin at 30 ppm, SEM = Standard error of the mean. WBCs = White blood cells, Hb = Hemoglobin, RBCs = Red blood cells, HCT = Hematocrit

Furthermore, a gradual yet statistically significant reduction in basophil concentration was observed in the PV group compared with the PP group. The lowest basophil concentration was recorded at 0.62% in the PV20 treatment group. However, this difference did not reach statistical significance when compared to the PV10 and PV30 treatments, which resulted in basophil concentrations of 3.53% and 1.78%, respectively. Conversely, the PN (7.30%) and PP (4.80%) treatments exhibited the highest basophil concentrations, which were significantly different from all PSV treatments (p = 0.014). While the PN treatment exhibited the highest basophil concentration, it did not demonstrate a statistically significant difference compared with the PP, PV10, and PV30 treatments (7.30% vs. 4.80%, 3.53%, and 1.78%, respectively). Other hematological parameters, including neutrophils, lymphocytes, monocytes, eosinophils, Hb, RBCs, HCT, and platelets, showed no significant differences among treatments.

### Histopathological examination

#### Liver

**Figure 3:**
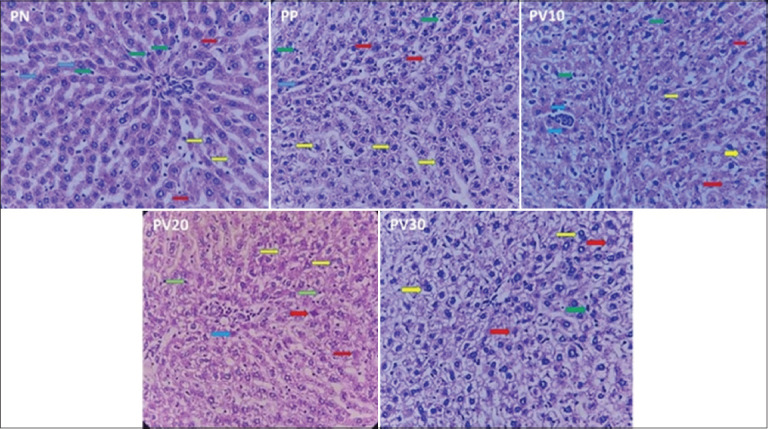
A representative photomicrograph (400×) of rat liver tissue stained with hematoxylin and eosin. Blue arrows indicate normal hepatocytes, red arrows indicate necrosis, yellow arrows indicate hydropic degeneration, and green arrows indicate inflammatory cells. PN = Negative control, PP = Positive control, PV10 = Phosvitin at 10 ppm, PV20 = Phosvitin at 20 ppm, PV30 = Phosvitin at 30 ppm.

Based on the histological examination of the liver and kidney using H&E staining, several morphological changes were observed in the samples analyzed. Normal hepatocyte cells were observed in the liver (Figure 3). However, necrosis, hydropic degeneration, and inflammatory cell infiltration were also observed.

Our findings from the histopathological examination of the liver (Table 5) indicate that the treatments did not significantly affect the number of hepatocytes. However, a notable discrepancy was observed in the number of inflammatory cells, with PV30 showing the highest count of 25.84, which was significantly different from PV10 (9.65) (p = 0.049). In contrast, PP (12.93) and PV20 (17.95) did not differ significantly from the other treatments. The necrosis score varied considerably among the treatments, with PV10 exhibiting the highest score of 4.00, significantly different from PN (1.00), PP (1.33), and PV20 (2.00) (p < 0.05). The necrosis scores for PV20 and PV30 (2.00 each) did not differ significantly from those of the other treatments (Table 5). Similarly, the hydropic degeneration score showed no significant differences between the treatments. Our results indicated that PSV administration at different doses affected the number of inflammatory cells and the necrosis score. However, it did not significantly impact the number of hepatocytes or the hydropic degeneration score.

**Table 5:** Effects of phosvitin on pathophysiological changes in the liver of test rats across different treatment groups.

Variable	Treatments					SEM	p-value

	PN	PP	PV10	PV20	PV30		
Hepatocytes	37.50	22.33	0.45	20.25	21.92	5.113	0.382
Inflammatory cells	18.60^ab^	12.93^ab^	9.65^b^	17.95^ab^	25.84^a^	1.691	0.049
Necrosis score	1.00^b^	1.33^b^	4.00^a^	2.00^b^	2.00^b^	0.266	0.035
Hydropic degeneration score	2.00	3.00	4.00	2.75	3.00	0.330	0.535

Different letters (a–c) within rows indicate significant differences between treatments (p < 0.05). PN = Negative control, PP = Positive control, PV10 = Phosvitin at 10 ppm, PV20 = Phosvitin at 20 ppm, PV30 = Phosvitin at 30 ppm, SEM = Standard error of the mean

#### Kidney

**Figure 4:**
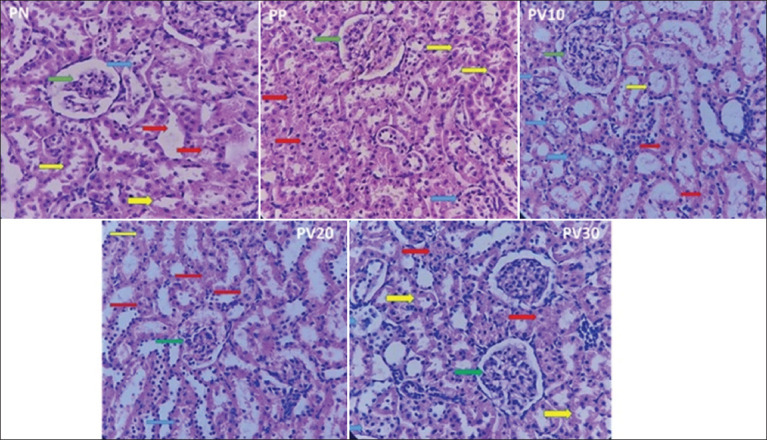
A representative photomicrograph (400×) of rat kidney tissue stained with hematoxylin and eosin. Green arrows indicate glomeruli, yellow arrows indicate normal tubules, red arrows indicate tubular necrosis, and blue arrows indicate tubular degeneration. PN = Negative control, PP = Positive control, PV10 = Phosvitin at 10 ppm, PV20 = Phosvitin at 20 ppm, PV30 = Phosvitin at 30 ppm.

Figure 4 shows that the kidney exhibits normal glomerular and tubular structures; however, regions of tubular necrosis and degeneration are also present. The research findings indicate that varying concentrations of PSV had a non-significant effect (p > 0.05) on the renal histological structure of test rats across groups, suggesting a threshold effect (Table 6). The evaluation was based on the number of normal tubules, tubular necrosis scores, and hydropic degeneration scores. The PV30 group had the highest mean number of normal tubules (23.40), whereas the PP group exhibited the lowest value (14.25). However, statistical analysis revealed that this difference was not significant (p = 0.096), although a trend suggested an increase in the number of normal tubules with higher PSV concentrations.

**Table 6:** Effects of phosvitin on pathophysiological changes in the kidney in test rats across different treatment groups.

Variable	Treatments					SEM	p-value

	PN	PP	PV10	PV20	PV30		
Normal tubule	15.50	14.25	20.56	18.38	23.40	1.057	0.096
Tubular necrosis score	1.00	1.86	1.00	1.75	1.60	0.249	0.801
Hydropic degeneration score	1.00	1.33	1.25	1.75	1.60	0.256	0.918

PN = Negative control, PP = Positive control, PV10 = Phosvitin at 10 ppm, PV20 = Phosvitin at 20 ppm, PV30 = Phosvitin at 30 ppm, SEM = Standard error of the mean

The assessment of kidney damage, based on tubular necrosis and hydropic degeneration scoring (Table 6), indicated that most treatment groups fell within the mild damage category (score 1), with no statistically significant variations (p > 0.05). The tubular necrosis score in the PP group (1.86) indicated a higher degree of kidney damage compared to the other groups. In contrast, PV10 and PN had the lowest scores (1.00). The groups treated with isolated PSV showed a reduction in necrosis scores compared with PP, although the difference was not statistically significant (p = 0.801). For hydropic degeneration, PV20 had the highest score (1.75), followed by PV30 (1.60), PP (1.33), PV10 (1.25), and PN (1.00). All hydropic degeneration scores remained within the mild damage category; however, the PV20 group displayed a trend toward more pronounced mild damage.

### Brain tissue heavy metal analysis

**Figure 5:**
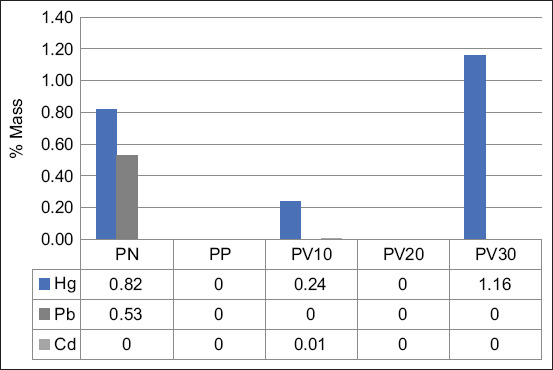
Concentration of mercury (Hg), lead (Pb), and cadmium (Cd) in rat brain tissue samples. PN = Negative control, PP = Positive control, PV10 = Phosvitin at 10 ppm, PV20 = Phosvitin at 20 ppm, PV30 = Phosvitin at 30 ppm.

Hg was detected in brain tissue samples from the PN, PV10, and PV30 groups, with respective mass percentages of 0.82%, 0.24%, and 1.16%, respectively. In contrast, no detectable Hg was found in the PP and PV20 groups. Lead (Pb) and cadmium (Cd) were present at significantly lower concentrations than Hg and only appeared in specific groups. Pb was detected in the PN group at a mass of 0.53%, whereas Cd was identified solely in the PV10 group at a mass percentage of 0.01% (Figure 5).

## DISCUSSION

### Isolation and characterization of PSV

#### Metal-binding and antioxidant mechanisms

PSV, a highly phosphorylated egg yolk protein, demonstrates strong metal-chelating activity owing to its abundance of phosphate groups, primarily on serine residues. These negatively charged groups enable the formation of stable mono-, bi-, or multidentate complexes with metal ions such as Fe^3+^, Ca^2+^, magnesium ion, and zinc ion [14, 15]. Multidentate coordination significantly enhances complex stability, thereby reducing the bioavailability of metal ions and limiting their oxidative reactivity [14]. This chelation mechanism not only facilitates mineral storage and transport within the yolk but also contributes to the antioxidant properties of PSV by suppressing the formation of metal-induced reactive oxygen species [29].

#### Biological functions of PSV

The biological functions of PSV, ranging from calcium binding for bone health [30] and the stimulation of osteoblast-related gene expressions [31] to antibacterial [32, 33], anti-aging [34], and antiviral activities [35–37], are closely tied to its structural features. Its high phosphorylation density is central to these effects, as it governs the charge distribution and metal-binding affinity of the protein [14]. PSV can bind up to 60 iron atoms per molecule, and its binding strength is directly related to the integrity of its phosphoserine clusters [21]. Hydrolysis alters the metal-binding profile of this structure, confirming that phosphorylation density is a key determinant of chelation efficacy [13, 21].

#### Food and nutritional applications

In food applications, PSV functions as a natural emulsifier and antioxidant, enhancing product stability and providing health benefits [13, 38]. Given its multifunctional properties and strong metal-binding potential, further research is warranted to explore its therapeutic applications, particularly in the detoxification of heavy metals. Understanding the relationship between its molecular structure and biological activity is essential for optimizing its use in nutraceuticals and environmental health interventions.

#### Experimental isolation and characterization

This study successfully isolated PSV from commercial egg yolk using the method described by Ko *et al*. [23], resulting in a product with 71% recovery and 51.68% purity. Although lower than that of commercial PSV (86.13%), the sample demonstrated comparable antioxidant activity (IC_50_ = 124.85 ppm vs. 125.27 ppm) (Table 1), confirming its functional potential [13, 14]. Figure 1 shows the spectral analysis results, which verified the similarity of the isolated product to the standard. However, SDS-PAGE revealed that standard PSV exhibited higher purity, as indicated by the number of protein bands (Figure 2). The presence of dominant protein bands with molecular weights ranging from 39.5 kDa to 38.5 kDa (Table 2) aligns with the findings of Anton *et al*. [39] and Toan *et al*. [40], who reported that PSV consists of multiple polypeptides with molecular weights ranging from 31 kDa to 41 kDa. Differences in protein and PSV contents may be due to variations in the separation and purification methods used during isolation and extraction [40]. The majority of non-protein components in the isolated sample are likely residual lipids or carbohydrates that were not completely removed during the extraction process [41].

#### Factors influencing extraction efficiency

The successful isolation of PSV is crucial in various biotechnological and nutritional applications. An efficient and reliable PSV extraction procedure depends on multiple factors, including the selection of chemical reagents, extraction conditions, and purification techniques [41–43]. Although the extraction procedure developed by Ko *et al*. [23] has proven to be effective, the results obtained in this study indicate a lower recovery rate and purity. One of the key factors that could influence the outcomes of PSV extraction is the quality and source of raw materials. Egg quality is significantly influenced by various factors, including chicken breed [44], feed composition [45], hen age [46], rearing system [47], and egg storage conditions [48]. Variations in egg composition may subsequently affect the composition of other egg proteins, including PSV.

#### Antioxidant properties of isolated PSV

Despite its lower purity, the isolated PSV retained strong antioxidant properties, which are attributable to its capacity to bind redox-active metals, such as ferrous and copper ions, thereby mitigating free radical formation and oxidative stress [13, 22]. These results support its potential as a cost-effective bioactive compound with wide-ranging applications in nutrition, medicine, and food technology.

### Hematological findings

#### Importance of hematological biomarkers

Hematological parameters are widely recognized as sensitive biomarkers for evaluating systemic toxicity. Blood reflects physiological and biochemical alterations in response to toxicant exposure, making it a valuable tool for the early detection of health disturbances [49]. MeHg, a potent neurotoxin, remains in the bloodstream for an extended period, making regular monitoring essential to track its impact [50]. Hematological profile changes often serve as the first signs of toxicity, allowing timely intervention to prevent severe health consequences [51]. Therefore, blood tests are a reliable means of assessing health risks and ensuring patient safety.

#### Effects of MeHg exposure

In this study, the rats exposed to MeHg by consuming contaminated fish showed an increase in WBC count (Table 3), suggesting immune activation likely due to oxidative stress or inflammation. This aligns with the findings of Hounkpatin *et al*. [51] and Innih and Nwachukwu [52]. Differential leukocyte analysis revealed a reduction in neutrophils and an increase in lymphocytes, indicating a shift toward adaptive immunity. Increased monocytes, eosinophils, and basophils suggest an immune response to Hg-induced stress.

The effects of Hg on other hematological parameters, such as RBC, Hb, and HCT, were relatively minor compared to WBC components (Table 3). A decrease in Hb levels was observed alongside increased RBC and HCT levels, which may reflect the body’s adaptation to Hg exposure. This adaptive response likely involves erythropoiesis stimulation to compensate for potential reductions in oxygenation. In addition, a decrease in platelet count may indicate possible disruptions in the blood coagulation system. The increase in RBC and HCT levels in this study differs from previously reported findings on Hg intoxication, particularly those involving mercuric chloride (HgCl_2_), which tend to show reductions in these indicators [51, 53]. This discrepancy may be attributed to differences in the toxicological properties of Hg compounds and variations in exposure duration and dosage, which influence the severity of Hg’s effects on tissues and organs [2, 54].

#### Toxicological differences in Hg forms

In this study, Hg was administered as MeHg to rats through gavage for 14 days, whereas other studies used inorganic Hg (HgCl_2_) with exposure durations ranging from 28 to 84 days. MeHg is an organic form of Hg that is more lipophilic than HgCl_2_ [54], allowing it to easily penetrate cell membranes and distribute throughout various tissues, including the central nervous system. MeHg disrupts normal cellular function by binding to sulfhydryl groups on proteins, leading to oxidative stress and inflammation. Oxidative stress further impairs the body’s ability to transport oxygen efficiently, potentially triggering hypoxic responses in specific tissues. The body stimulates erythrocyte production to enhance oxygen transport capacity in the bloodstream as a physiological adaptation to hypoxia. This may explain the increased RBC and HCT levels observed in rats exposed to MeHg for a short duration.

#### Pathways of Hg exposure

Hg exposure occurs through various pathways, including consumption of seafood, occupational hazards, and environmental pollution. Dietary intake, particularly from seafood, remains the primary source, as MeHg accumulates in fish and shellfish, especially in predatory species at the top of the food chain [55, 56]. Occupational exposure is prevalent among ASGM, where Hg is used to extract gold, contaminating the air, water, and soil, thereby endangering workers and nearby communities [55]. Exposure occurs through the inhalation of Hg vapor or through contact with contaminated water, leading to severe health risks. Environmental pollution significantly intensifies Hg exposure, as Hg enters water bodies through natural processes and human activities, such as mining and industrial pollution. This contamination threatens aquatic ecosystems, human health, and livestock, particularly when consuming Hg-laden fish and livestock [57, 58].

#### Global health implications and mitigation strategies

The impact of Hg exposure is a critical global health issue, underscoring the urgent need for targeted interventions to reduce exposure levels, contamination, and long-term health risks in humans and livestock [8, 56]. Several strategies can be employed to mitigate the health and environmental effects of Hg exposure, including strengthening regulations and policies to limit Hg emissions from industries such as mining and coal-fired power plants [57, 58], enhancing public awareness and educational initiatives [59], conducting environmental remediation efforts [58], and implementing nutritional interventions [8, 60].

#### Effect of PSV treatment

In this study, a 4-week intervention with PSV, known for its strong metal-binding activity [9], significantly reduced WBC counts, with the lowest WBC count observed in the PV30 group (Table 4). This indicates that PSV may help regulate immune responses, reduce inflammation, and reduce the immune stress caused by Hg exposure. A notable effect was also observed in basophil levels, which decreased across all PSV doses, with PV20 showing the lowest counts. Despite these changes in WBC count and basophil count, PSV supplementation did not significantly affect other hematological parameters, including neutrophil count, lymphocyte count, monocyte count, eosinophil count, Hb concentration, RBC count, HCT, and platelet count. These findings suggest that PSV can modulate specific aspects of the immune system without altering hematological function profiles.

#### Mechanisms of Hg-induced hematological effects

The hematological effects of Hg, as detailed in the systematic review by Vianna *et al*. [61], can arise through multiple pathways, including direct toxicity to bone marrow, hypersensitivity and immune responses, chronic apoptosis, autoimmune reactions, inflammation, hemolysis, and even blood loss. WBCs, including basophils, play a crucial role in the immune response triggered by infections or harmful substances. However, Hg can sometimes induce an excessive or dysregulated immune response, leading to persistent inflammation. This condition results in elevated WBC and basophil levels, indicating an excessive inflammatory state in the body. Interestingly, PSV may help regulate or even suppress this heightened immune response by inhibiting Hg-induced pro-inflammatory pathways, contributing to the reduction of excessive WBC and basophil production.

### Histopathology of the liver and kidney

#### Liver

The liver, kidneys, digestive system, and central nervous system are the primary targets of Hg toxicity [62]. Histopathological analysis of liver tissue from Hg-exposed rats, following PSV supplementation (Figure 3), revealed the presence of normal hepatocytes; however, the inflammatory cell count and necrosis scores varied. As shown in Table 5, the PV30 group exhibited the highest number of inflammatory cells, showing a significant difference compared to the PV10 group (p = 0.049). The highest necrosis score was recorded in PV10, which differed significantly from PN, PP, and PV20 (p < 0.05). In contrast, hydropic degeneration scores did not differ significantly across treatments. The presence of inflammatory cells in liver tissue is often associated with immune responses triggered by oxidative stress and toxic exposure [62]. Hepatocyte necrosis can be initiated by various mechanisms, including apoptosis and necroptosis, due to the excessive accumulation of reactive oxygen species [63]. Other studies have suggested that proteins such as PSV possess strong antioxidant properties, which may potentially reduce inflammation and oxidative stress in liver tissue [64].

#### Kidney

The increase in normal tubules (Table 6) observed in the PV30 group suggests the potential anti-inflammatory and antioxidant activity of PSV in protecting kidney structures from oxidative stress-induced damage. However, the scores for hydropic degeneration and tubular necrosis in PV20 and PV30 exhibited a slight increase compared to PV10, indicating that the protective effects of PSV may reach an optimal threshold at specific doses, beyond which additional supplementation could disrupt cellular homeostasis. This aligns with the findings of Rajashekar [65], who suggested that at high concentrations, antioxidants may act as pro-oxidants, disturbing redox balance and potentially triggering secondary oxidative stress. In addition, the accumulation of hydropic degeneration in PV20 and PV30 may reflect oxidative stress-induced cellular metabolic disturbances or mitochondrial dysfunction [66]. Pharmacokinetic modeling is necessary to understand how Hg is absorbed, distributed, and retained at various PSV doses.

Based on histopathological analysis of the liver and kidneys, this study suggests that PSV affects inflammatory cell counts and necrosis scores in liver tissue, but does not significantly impact hepatocyte numbers or the degree of hydropic degeneration. Its effects on the kidneys appear more subtle, showing a trend toward protection against tubular necrosis that has yet to reach statistical significance. These differences may arise from variations in the protective mechanisms provided by PSV across different tissue types. Further research is necessary to explore its role in reducing oxidative stress and enhancing organs’ regenerative capacity.

### Concentration of Hg in brain tissue

#### Heavy metal accumulation and environmental relevance

Exposure to heavy metals, particularly Hg, Pb, and Cd, has been a significant concern in toxicological research because of their impact on the nervous system [2]. Studies have documented substantial heavy metal pollution in various aquatic environments throughout Indonesia [67–70]. The presence of these metals in marine organisms, such as fish, snails, and green mussels, indicates bioaccumulation and biomagnification, posing health risks through the consumption of seafood.

#### Experimental findings

The analysis of Hg, Pb, and Cd concentrations in brain tissue samples from test rats (Figure 5) revealed variations in Hg levels, with Hg being the predominant contaminant. The highest Hg concentrations were observed in the PV30 and PN groups. Interestingly, PSV supplementation in the PV10, PV20, and PP groups reduced Hg accumulation (% mass) in rat brain tissue. Meanwhile, Pb and Cd were detected at much lower concentrations, appearing only in a few groups, suggesting minimal contamination from these metals. This finding underscores the potential of PSV in mitigating Hg accumulation in neural tissues, highlighting its role in counteracting heavy metal-induced toxicity.

#### Mechanistic considerations

This study suggests that PSV may influence Hg accumulation in brain tissue, with lower concentrations (PV10 and PV20) reducing Hg levels, whereas higher concentrations (PV30) seem to increase its presence. This indicates a complex interaction between PSV and Hg bioavailability, possibly related to its metal-chelating properties. Chelation therapy is a widely recognized strategy for reducing heavy metal toxicity, including Hg exposure. The toxic effects of Hg are primarily attributed to its high affinity for thiol (–SH) groups in proteins and enzymes, which disrupt cellular redox balance and induce oxidative stress, ultimately leading to tissue damage [9]. PSV, known for its strong affinity for metal ions due to its high phosphorylation [13, 14], may interact with Hg in a way that alters its distribution and accumulation. However, the increased Hg levels at higher PSV concentrations suggest that its chelation mechanism may not be straightforward and could involve additional biochemical pathways. Further research is necessary to determine whether PSV directly binds to Hg in a manner that facilitates its elimination or if it might unintentionally promote Hg retention under certain conditions. This study did not measure Hg excretion in urine or feces, which limits the ability to confirm systemic detoxification. Understanding these interactions is crucial for assessing the role of PSV in heavy metal removal and its potential for treating Hg-induced neurotoxicity.

#### Practical implications and one health perspective

The strong negative charge of PSV and its high affinity for divalent cations highlight its potential as a dietary component to lower the bioavailability of Hg. This metal-binding property supports its use in nutritional strategies aimed at reducing Hg exposure in humans and animals while benefiting the environment. The incorporation of PSV into food and feed systems aligns with the principles of the One Health approach. To practically realize this potential, rigorous regulatory assessment is essential. Toxicological testing, allergenicity screening, and dose optimization are crucial for ensuring safety, effectiveness, and compliance. The careful validation of PSV-based interventions will help them to be implemented across different species and build public trust in sustainable Hg remediation approaches.

## CONCLUSION

This study demonstrates that PSV isolated from egg yolk layers exhibits high recovery, moderate purity, and antioxidant activity comparable to commercial standards. *In vivo* supplementation, especially at 20 ppm, consistently reduced Hg-induced liver and kidney damage, decreased Hg buildup in brain tissue, and improved blood parameters. These results indicate a dose-dependent therapeutic effect, likely through mechanisms of chelation or metal redistribution. Although no obvious adverse effects were observed at the best dose, further safety testing is necessary for future use. The dual function of PSV as an antioxidant and metal chelator highlights its potential as a dietary therapeutic in Hg-prone populations.

To the best of our knowledge, this is the first study to demonstrate the chelating ability of egg yolk-derived PSV against Hg toxicity *in vivo*, highlighting its potential as a dietary detoxifying agent. In areas with high seafood consumption and increased Hg exposure, the antioxidant and metal-binding properties of PSV may provide protective benefits for both humans and livestock. Its impact on immune and tissue responses indicates its potential usefulness in therapeutic and preventive health strategies.

The integrated approach of this study, which combines chemical characterization, antioxidant assays, hematological profiling, histopathological evaluation, and tissue metal analysis, is a major strength. This comprehensive methodology provides a clear understanding of the biological effects of PSV and links data to physiological outcomes in a relevant animal model.

However, several limitations need to be acknowledged. The isolated PSV showed lower purity than its commercial counterpart, which could impact reproducibility and consistency. The animal study was limited to a single supplementation period and a relatively small sample size, which may have reduced the statistical power and generalizability of the findings. Although some trends were observed, certain outcomes, especially in kidney histopathology, did not reach statistical significance. Reductions in brain Hg levels were observed; however, the extent and consistency of these effects remain uncertain. These limitations may be due to the short treatment duration, despite multiple dosage levels being tested. In addition, no long-term follow-up was conducted to assess Hg excretion, organ regeneration, or survival beyond the 4 weeks. Tissue-specific responses to PSV require further investigation to better understand its varying effects across different organ systems.

Future research should focus on optimizing extraction and purification methods to increase PSV yield and purity. More *in vivo* studies with different dosing regimens and treatment durations are necessary to establish the therapeutic threshold and safety profile of the PSV. Examining its effectiveness against other heavy metals across various organ systems and species, along with understanding its molecular mechanisms of action, will be crucial for advancing its translational potential.

In conclusion, this study contributes to the growing evidence supporting the therapeutic role of PSV in heavy metal detoxification. The results highlight its significance in toxicology and nutrition and lay the groundwork for future applications in food safety and public health.

## AUTHORS’ CONTRIBUTIONS

MS and SND: Developed and implemented a research framework. MS and IGASA: Data collection and supervised all research stages. CPD, AE, AR, and RSP: Isolation and characterization processes and *in vivo* study. MS and RAP: Analyzed and visualized the data. MS, IGASA, RSP, and RAP: Drafted the original version of the manuscript. MS, RSP, CPD, AE, SND, and RAP: Reviewed and revised the manuscript. All authors have read and approved the final version of the manuscript.
